# Soil-Plant Metal Relations in *Panax notoginseng*: An Ecosystem Health Risk Assessment

**DOI:** 10.3390/ijerph13111089

**Published:** 2016-11-05

**Authors:** Xiaohong Ou, Li Wang, Lanping Guo, Xiuming Cui, Dahui Liu, Ye Yang

**Affiliations:** 1 College of Resources and Environment, Huazhong Agricultural University, Wuhan 430070, China; ogh1986@163.com; 2 Kunming Key Laboratory of Sustainable Development and Utilization of Famous-Region Drug, Key Laboratory of *Panax notoginseng* Resources Sustainable Development and Utilization of State Administration of Traditional Chinese Medicine, Faculty of Life Science and Technology, Kunming University of Science and Technology, Kunming 650500, China; sanqi37@vip.sina.com; 3 Institute of Medicinal Plants, Yunnan Academy of Agricultural Sciences, Kunming 650205, China; Lwang20@163.com; 4 Chinese Medica Resources Center, China Academy of Chinese Medicinal Sciences, Beijing 100700, China; glp01@126.com; 5 College of Pharmacy, Hubei University of Chinese Medicine, Wuhan 430065, China

**Keywords:** *Panax notoginseng* (Burk) F. H. Chen, heavy metal, Chinese herb medical, risk

## Abstract

This study features a survey of the content of heavy metals (Pb, Cd, Cr, As, Hg and Cu) in root and cultivation soils of *Panax notoginseng* (*P. notoginseng*), carried out in China’s Yunnan Province. The average contents of Pb, Cd, Cr, As, Hg, and Cu in the soil were 61.6, 0.4, 102.4, 57.1, 0.3, and 35.1 mg·kg^−1^, respectively. The heavy metals’ pollution indexes can be ranked as follows: As > Cd > Hg > Cu > Cr > Pb. The proportion of soil samples at slight, middle, strong, very strong, and extremely strong levels of potential environmental risk had values of 5.41%, 21.62%, 35.14%, 10.81%, and 27.03%, respectively. The potential environment risk index (*RI*) showed that 29.73% out of the total sample sites were above the level of strong and extremely strong. The ranges of Pb, Cd, Cr, As, Hg, and Cu content in tuber were 0.04–3.26, 0.04–0.33, 0.22–5.4, 0.10–1.8, 0.00–0.02, and 5.0–20.9 mg·kg^−1^, respectively. In combination with *P. notoginseng* consumption data, the estimated heavy metal daily intakes (EDIs) were 0.08–0.23, 0.006–0.019, 0.17–0.52, 0.04–0.12, 0.001–0.002, and 0.59–1.77 μg·kg^−1^·bw/day. All target hazard quotients (THQs) of individual elements and hazard indexes (*HI*) were less than one. The present study indicates that most of the *P. notoginseng* cultivation soil in the province of Yunnan presented slight and moderate ecological risk. Thus, more attention should be given to the heavy metals As, Cd, and Hg when selecting planting areas for the cultivation of *P. notoginseng*. Health risks associated with the intake of a single element or consumption of the combined metals through *P. notoginseng* are absent.

## 1. Introduction

Radix et Rhizoma Notoginseng is the dry rhizome of *Panax notoginseng* (Burk) F. H. Chen (*P. notoginseng*) of the *Araliaceae* ginseng species. It is a precious traditional herb in China, which possesses the function of dissipating blood stasis and arresting bleeding, thereby promoting the subsidence of swelling and relieving pain. The world famous Chinese patent medicine *Yunnan White Drug* and *Pianzihuang* both use *P. notoginseng* as their primary raw material. Therefore, the demand for *P. notoginseng* in the global medicine market is increasing yearly.

In recent years, severe heavy metal pollution of *P. notoginseng* cultivation regions has attracted much attention from consumers and regulators. The pollution is mainly a result of high soil background values, mining activities, and the application of pesticides that contain heavy metals. Heavy metals in the soil directly influence the safe production and use of traditional Chinese medicine [1]. Intake of herbs polluted by heavy metals could lead to a series of acute and chronic poisoning reactions [2,3]. The tuber of *P. notoginseng* is the main medicinal part, and it possesses a strong heavy metal accumulation capacity, especially for Cd and As. Thus, events where the rhizome of *P. notoginseng* contains amounts above the average heavy metal content induced by soil pollution have been reported occasionally [4,5,6]. Thus, we must pay more attention to securing the safety of cultivation soil, and preventing the above average presence of heavy metals in *P. notoginseng* and corresponding products.

*P. notoginseng* cultivation soil may accumulate elevated levels of potentially toxic elements (PTEs) from both point and diffuse sources of pollution in Wenshan State and/or Yunnan Province [7,8]. Wenshan State is one of the main production areas of metal minerals (Sn, Zn, Mn, Sb, W, Al) in Yunnan Province. Abundant reserves of nonferrous metals lead to high levels of heavy metals in the arable land [9]. In addition, human activities (such as industrialization, traffic, dometic sewage, atmospheric deposition, and so on) exacerbate the pollution of arable soil [10,11]. Pollutants in soil represent a continuing risk to the urban ecological system, especially human health [12,13]. Prior research shows that herbs constitute an important link in the transfer of heavy metal from soil to man [14]. Intake of herbs polluted by heavy metals could cause a series of acute and chronic poisoning reactions [2]. Therefore, it is important to increase research on heavy metal pollution and risk assessments of *P. notoginseng’s* cultivation soil and products.

Non-cancer risk assessments are typically conducted to estimate the potential health risks of pollutants using the target hazard quotient (THQ), a ratio of the estimated dose of a contaminant to the dose level below which there will be no appreciable risk [15]. To assess the overall potential risk for non-carcinogenic effects posed by more than one element, the hazard index (*HI*) approach has been developed by the U.S. Environmental Protection Agency (USEPA) [16]. This approach assumes that simultaneous sub-threshold exposures to several chemicals could result in an adverse health effects, and that the magnitude of the adverse effect will be proportional to the sum of the ratios of the sub-threshold exposures to acceptable exposures.

In order to guarantee the safety of medicinal products, we conducted a pollution status investigation and evaluation for heavy metals in *P. notoginseng* cultivation soil and raw materials. Acquiring data from the producing areas of *P. notoginseng* enabled us to conduct an assessment of both cultivation and health risks.

## 2. Materials and Methods

### 2.1. Sample Collection

**Figure 1:**
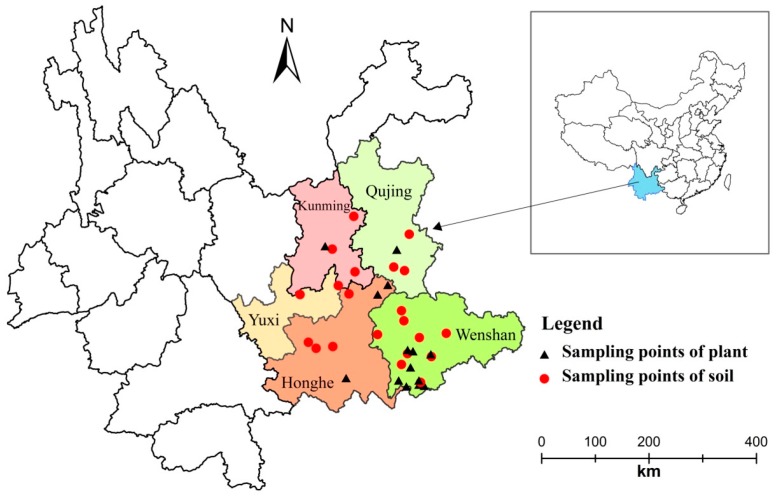
Sampling points of soils and plants (created by the *ArcGIS 10.2* program).

We selected a genuine producing area of *P. notoginseng* in the Yunnan Province as our study area. Table 1 and Figure 1 show details of the sample sites and sampling points. The latitude is from N, 22.88° to N, 25.73°; longitude is from E, 102.43° to E, 104.88°; and the altitude is from 762 m to 2114 m.

Our sampling period took place from 18 October to 26 November 2013. We investigated 37 cultivation regions, 37 soil samples, and 22 plant samples. For soil sampling, we collected 0–20 cm soil from each cultivation region using a five-point method: the soil was mixed adequately and subsampled, then air-dried and finely ground, and finally prepared for heavy metal content determination. For plant sampling, we randomly collected 20 plants from three-year old *P. notoginseng* that would be harvested as a commodity. We then washed and dried these according to commercial instructions, then we separated the whole root from the rhizome and hair root (the three parts used according to the Chinese Pharmacopoeia), and then we collected the rhizome as our plant samples for this study. We finely ground and subsampled the rhizome samples for heavy metal content determination.

### 2.2. Detection of Heavy Metals

We detected heavy metals (Pb, Cd, Cr, As, Hg and Cu) in soil and plant samples via the methods GB 15618-1995 [17] and WM/T2-2004 [18], with slight modifications. For heavy metal detection in the soil, we digested 0.25 g of soil samples with 10 mL HCl and 0.5 mL HClO_4_, and then diluted the mixture with 10 mL distilled water. Finally, we prepared the mixture for determination. We replicated the process three times for each sample.

For Pb, Cd, Cr, and Hg, we digested 2.00 g plant samples at 120 °C for 3.5 h with 3 mL HNO_3_ and 2 mL H_2_O_2_. After cooling, we diluted the digested solution with 10 mL distilled water, and then prepared it for determination. For As, we digested 5.00 g plant samples with 10 mL HNO_3_-HClO_4_ (4:1), and 10 mL H_2_SO_4_. We then cooled the mixture and diluted it with 50 mL distilled water, finally preparing it for determination. For Cu, we digested 1.00 g plant samples with 5 mL HNO_3_, then diluted the digestive solution with 10 mL distilled water, and finally prepared it for determination. We replicated the process three times for each sample.

We determined Pb, Cd, Cr, and Cu through an atomic absorption spectrophotometer (PEAA800, Perkin Elmer Inc., Waltham, WA, USA). Detection limits were Pb 15 mg·L^−1^, Cd 0.8 mg mg·L^−1^, Cr 3 mg·L^−1^, and Cu 1.5 mg·L^−1^, respectively. We detected As and Hg via hydride generation automatic fluorescence (HG-AFS 9230, Jitian Co., Beijing, China), and detection limits were As < 0.01 μg·L^−1^ and Hg < 0.001 μg·L^−1^.

### 2.3. Soil Heavy Metal Pollution and Potential Environmental Risk Assessment

#### 2.3.1. Soil Heavy Metal Pollution and Quality Assessment

We used the pollution index (*P_i_*) in this study. We measured the quality of soil environment classification using a single factor contaminant index and the comprehensive pollution index method. The single factor contaminant index calculation formula was as follows: single factor contaminant index:

(1) $$P_{i}=\frac{C_{i}}{S_{i}}$$

where *P_i_* is the pollution index; *C_i_* and *S_i_* represent the heavy metal (*i*) concentrations in the soil and evaluation standard values (GB15618-1995, Grade 2 for soil) [17], respectively. We evaluated soil quality by referencing to the state’s soil environment quality standards (see Table 2).

The Newmerow composite index method not only takes account of all the individual evaluation factors, but also highlights the importance of the most contaminated elements. Our comprehensive pollution index calculation formula was as follows: comprehensive pollution index:

(2) $$P_{c}=\sqrt{\frac{{{\left(\frac{C_{i}}{S_{i}}\right)}^{2}}_{mean}+{{\left(\frac{C_{i}}{S_{i}}\right)}^{2}}_{max}}{2}}$$

*P_c_* is comprehensive pollution index; ${\left(\frac{C_{i}}{S_{i}}\right)}_{mean}$ and ${\left(\frac{C_{i}}{S_{i}}\right)}_{max}$ are the mean and maximum values of single factor contaminant index, respectively. Table 2 shows the classification standards of soil heavy metal pollution evaluation.

#### 2.3.2. Soil Heavy Metal Potential Environmental Risk Assessment

In 1980, Hankanson proposed a potential environmental risk assessment [19]. The comprehensive potential environment risk index calculation formula was as follows:

(3) $$RI=\sum_{k=0}^{n}E_{r}^{i}=\sum_{k=0}^{n}T_{r}^{i}\times \frac{C_{s}^{i}}{C_{n}^{i}}$$

where *RI* is the comprehensive potential environment risk index; $E_{r}^{i}$ is the potential ecological risk factor of a single metal; $T_{r}^{i}$ represents for a certain kind of metal (*i*) toxicity response coefficient (Table 3) [20]; $C_{s}^{i}$—the measured value concentration of heavy metals (*i*) in the soil’s surface; and $C_{n}^{i}$—the reference background content of Yunnan Province soil (Table 3) [21].

### 2.4. Health Risk Assessment of Heavy Metal through P. notoginseng Consumption

#### 2.4.1. Estimated Daily Intakes (EDIs)

The estimated daily intakes (EDIs) of heavy metal (Pb, Cd, Cr, As, Hg, and Cu) depend on both the concentrations of heavy metal in *P. notoginseng* and consumption levels. The EDI of each heavy metal was determined by the following equation:

(4) $$\mathrm{EDI}=\frac{E_{F}\times E_{D}\times F_{\mathrm{IR}}\times C}{W_{\mathrm{AB}}\times T_{A}}$$

E_F_ is exposure frequency (365 days·year^−1^); E_D_ is exposure duration (40 years, from age 30 to age 70 (equal to the average lifetime)); F_IR_ is *P. notoginseng* ingestion rate (3–9 g·person^−1^·day^−1^) [22]. C is heavy metal concentration in *P. notoginseng* (mg·g^−1^); W_AB_ is average body weight (60 kg was adopted in the present study); and T_A_ is average exposure time for non-carcinogens (365 days·year^−1^ × number of exposure years, assuming 40 years).

#### 2.4.2. Target Hazard Quotient (THQ)

We assessed the health risk through consumption of *P. notoginseng* based on the target hazard quotient (THQ). A methodology for estimating the THQ was described in detail by USEPA [23]. We can assess THQ for residents through consumption of *P. notoginseng* by comparison with the provisional tolerable weekly intake (PTWI) for each element. The health risks were separately considered, since the contact pathway with each exposure medium changes with age. In this respect, the THQ is determined based on the methods modified from Chien et al. [24] by the following equation:

(5) $$\mathrm{THQ}=\frac{\mathrm{EDI}\times 7}{\mathrm{PTWI}}$$

Tolerable intake values of heavy metals, called PTWI, are set by the Food and Agriculture Organization/World Health Organization (FAO/WHO) [25]. PTWI is the maximum amount of a contaminant to which a person can be exposed weekly over a lifetime without an unacceptable risk of negative health effects. Intake estimates were expressed as per unit body weight (μg·kg^−1^·bw·week^−1^). The applied PTWI for Pb, Cd, Cr, As, Hg, and Cu were 25 μg·Pb·kg^−1^ bw·week^−1^, 7 μg·Cd·kg^−1^·bw·week^−1^, 6.7 μg·Cr·kg^−1^·bw·week^−1^, 350 μg·As·kg^−1^·bw·week^−1^, 4 μg·Hg·kg^−1^·bw·week^−1^, and 3500 μg·Cu·kg^−1^·bw·week^−1^ [25]. When THQ < 1, we assume that the effects of heavy metals ingested by humans are not obviously damaging to the body’s health.

#### 2.4.3. Hazard Index (*HI*)

Harrison and Chirgawi [26] reported that exposure to two or more pollutants may result in additive and/or interactive effects. Assuming the additive effects, THQs can be summed across constituents to generate a hazard index (*HI*) for a specific receptor-pathway combination. In this way, the potential risk of adverse health effects from a mixture of heavy metals in *P. notoginseng* can be calculated. We calculated the *HI* values through consumption of *P. notoginseng* for human beings as follows:

(6) $$HI=\overset{i}{\underset{n=1}{\sum }}\mathrm{THQn}$$

### 2.5. Statistical Analysis

We used DPS v7.05 software (Hangzhou Ruifeng Information Technology Co., Ltd., Hangzhou, China) for descriptive statistics, and used the ArcGIS 10.2 program (Esri China Information Technology Co., Ltd., Beijing, China) for producing spatial distribution maps by Kriging Geo-statistical Analysis.

## 3. Results and Discussion

### 3.1. Characteristics of Heavy Metal Content in Different P. notoginseng Cultivation Region

Yunnan Province is one of the main metal mineral (Sn, Zn, Mn, Sb, W, Al) production areas in China. Abundant reserves of nonferrous metals induce high levels of heavy metals in arable land [9,12]. Liu et al. even concluded that the above-average contents of Cu, As, and Hg in *P. notoginseng* cultivation soil are a result of mother-material from the soil [27]. However, so far the heavy metal content of *P. notoginseng* cultivation system still lacks systematic investigation and study in the Yunnan province. The present study therefore investigates a wider scope of heavy metal (Pb, Cd, Cr, As, Hg and Cu) content in soil and *P. notoginseng* plants.

**Figure 2:**
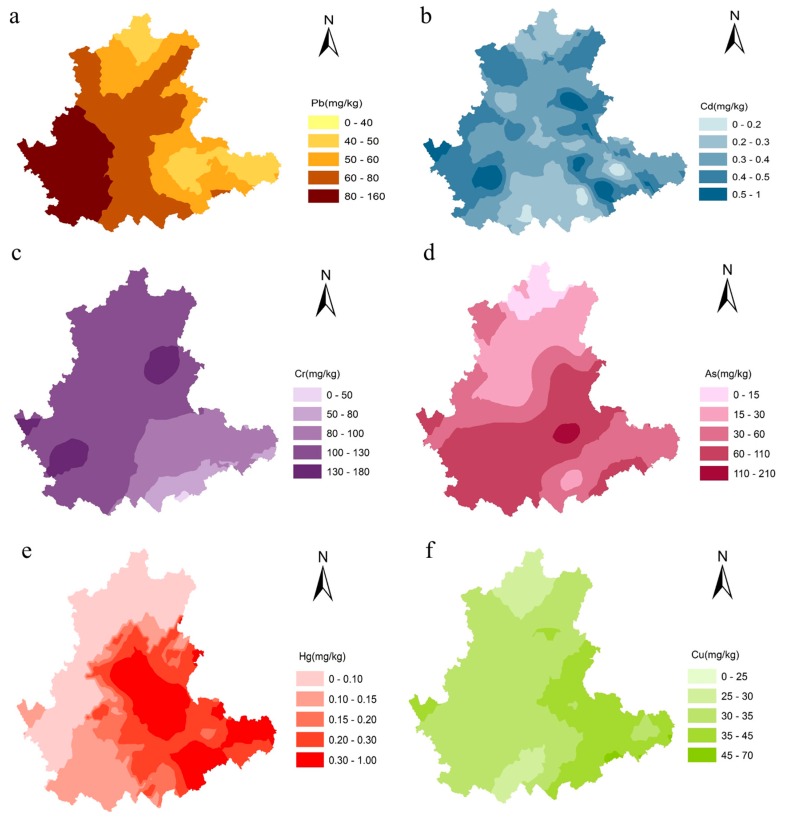
Spatial distribution maps of Pb (**a**); Cd (**b**); Cr (**c**); As (**d**); Hg (**e**) and Cu (**f**) in top soil in the survey area(*created by Geo-statistical Analysis Kriging of ArcGIS 10.2 program*).

Table 4 and Table 5 show the heavy metal content of the soil samples, as well as the degree to which safety standards are exceeded. Soil pH ranged from 4.58 to 6.37 in all the sample sites in the present study. We considered environmental quality standard for our soil samples [17]. The average contents of Pb, Cd, Cr, As, Hg, and Cu were 61.6, 0.4, 102.4, 57.1, 0.27, and 35.1 mg·kg^−1^, respectively. A comparison of the soil samples with the safety standard GB 15618-1995 showed that five (Cd, Cr, As, Hg, and Cu) of the six heavy metals exceeded the standard by more than the other soil samples. In the study area, the soil Pb content’s spatial distribution pattern diminished from west to east, with the highest Pb concentration found around the Yuxi area (Figure 2a). However, no sample soil exceeded the safety standard (Table 4 and Table 5). In thirteen soil samples, the Cd content exceeded the standard by a percentage of 35.14% (Table 4 and Table 5). The spatial distribution map of Figure 2b illustrates that the Cd content in the northern and southern areas were lower than the safety standard, but were higher in the central areas. Most of the study areas had higher Cr content, which diminished from northwest to southeast (Figure 2c). However, only seven samples accounted for 18.9% of amounts above the safety standard (Table 4 and Table 5). The highest soil Cr content was around the Qujing and Honghe areas (Figure 2c). The amount by which the As content exceeded the safety standard was 23 times, accounting for 62.2% (Table 4 and Table 5). The spatial distribution of the As content in the topsoil decreased in areas away from the central area, and the highest As content area was around Qiubei (Figure 2d). The results of As distribution were consistent with Zu et al. and the reason for this is the mining of arsenic minerals around the Qiubei area [28]. Eleven soil samples accounted for 29.7% of the amounts above the Hg safety standard (Table 4 and Table 5), and most above-average sites were distributed in the northeastern of the study area, especially around Qujing, Wenshan, Qiubei, and Xichou (Figure 2e). The spatial distribution map of Cu showed that a small area in the southeastern was above the safety standard (Figure 2f). The number of samples exceeding the safety standard was three, which accounted for 8.1% (Table 4 and Table 5). The number of sample sites that had above-average amounts of each heavy metal can be organized as follows: As > Cd > Hg > Cr > Cu. Thus, it can be seen that most of the *P. notoginseng* cultivation soil was polluted by As, Cd, and Hg. These survey results are inconsistent with the research of Chen et al. on Cd, Cr, Cu, and Pb [29], Lin et al. on As [30], and Yan et al. on Hg, As, Cd, and Cr in general [1]. The above-average rates mentioned in the literature are 48%–66.7% for As, 60%–75% for Cd, 73.7% for Hg, and all prior investigations indicate that the *P. notoginseng* cultivation soil is free from Pb contaminate. Therefore, more attention should be paid to As, Cd, and Hg when selecting land for the cultivation of *P. notoginseng*.

### 3.2. Pollution Status and Potential Environment Risk of P. notoginseng Cultivation Soil

The single factor pollution index and comprehensive pollution index are widely adopted when assessing the heavy metal pollution status of soil [31,32]. We firstly assessed the heavy metal pollution status of *P. notoginseng* cultivation soil by *P_i_* and *P_c_*. The single heavy metal pollution index of Cd, Cr, As, Hg, and Cu indicated that the soil was slightly over-polluted with all of these heavy metals. The pollution index of each heavy metal was in the order of As > Cd > Hg > Cu > Cr > Pb (Table 6). 35.1%, 18.9%, 18.9%, 13.5%, and 8.1% of soil samples were slightly polluted by As, Cd, Cr, Hg, and Cu. 21.6%, 13.5%, and 13.5% of soil samples were medium-polluted by As, Cd, and Hg. 5.4%, 2.7%, and 2.7% of soil samples were heavily polluted by As, Cd, and Hg, respectively. The range of comprehensive pollution index was 0.6–4.1, and the average value was 1.5. Twenty-six samples were over the slightly polluted level, with a pollution proportion accounting for 70.3%. The higher *P_c_* of heavy metals in *P. notoginseng* cultivation soil was mainly induced by the high *P_i_* of As. Only 18.9% of the samples’ *P_c_* values were lower than 0.7 (safety degree). Thus, it can be seen that most of the *P. notoginseng* cultivation soil samples were polluted by Cd, Cr, As, Hg, and Cu (especially As, Cd, and Hg). This is consistent with the results of Wang and Yan [8], whose results indicate that the contents of As, Hg, and Cd in the soil are high *P_i_* in Yunnan Province.

### 3.3. Environmental Risk Assessment of Heavy Metals in P. notoginseng Cultivation Soil

We used a pollution index to assess the heavy metal content of heavy metal, but the same index cannot be used to indicate the toxic risk degree. Hankanson’s potential environmental risk assessment mainly focuses on the toxic degree of heavy metals to the environment and humans. It consists of a single metal potential environment risk index $\left(E_{r}^{i}\right)$ and a comprehensive potential environment risk index (*RI*). This evaluation method reflects the biological effectiveness, relative contribution, and geographic and spatial difference that form the index that comprehensively reflects the effects of heavy metals on the environment. We used this method to detect the potential environmental risk levels of heavy metals in *P. notoginseng* cultivation soil.

Table 7 shows the single metal potential environmental risk index $\left(E_{r}^{i}\right)$ classification of *P. notoginseng* cultivation soil samples. We can see that all 37 soil samples had low potential for environment risk of Pb, Cr, and Cu. Less consideration might be given to these elements when *P. notoginseng* producers select their cultivation regions. The $E_{r}^{i}$ values of Cd for nine soil samples were at middle level, while four soil samples were at a strong level. Meanwhile, the $E_{r}^{i}$ values of As for fourteen soil samples were at middle level, and at a strong level with one sample. The proportion of Cd and As $E_{r}^{i}$ values that exceeded the middle level were 35.1% and 40.5%, respectively. These elements must therefore be considered when looking for cultivation land. The most serious pollution content for all soil samples was with Hg. The number of soil samples with $E_{r}^{i}$ values of Hg that exceeded the middle level was 35, accounting for 73%. Therefore, land that will be used for *P. notoginseng* cultivation must be clearly separated from regions contaminated by Hg.

The $E_{r}^{i}$ mean values of the six kinds of heavy metal we studied followed this order: Hg > Cd > As > Pb > Cu > Cr. This was extremely different from the average value order of *P_i_* As > Cd > Hg > Cu > Cr > Pb (Table 6). This is due to the toxicity response coefficient of heavy metals. Higher $T_{r}^{i}$ values induced heavy metals with relative low (Hg, Cd, As and Pb, Cu, Cr) *P_i_* values to increase. Numerous studies have reported similar results [33,34,35]. Our results indicate that toxicity response coefficients played an important role in assessing the $E_{r}^{i}$ values, and that Hg, Cd, and As indicated a considerable level of risk.

Table 8 shows the regional potential environment risk index (*RI*) classification of *P. notoginseng* cultivation soil. The range of *RI* was 95.8–895.1, with an average value of 318.7. Numbers of sample sites at the four *RI* degrees were 10, 16, six and five, respectively. The proportion of each risk degree was in the order of moderate > slight > strong > extremely strong. Sample site numbers above the level of strong and extremely strong were six and five, respectively (with 29.7% total). This proves that most of the *P. notoginseng* cultivation soil in the Yunnan province exhibits slight and moderate ecological risk. *RI* represents the sum $E_{r}^{i}$ value of each heavy metal. Hg, Cd, and As are big contributors to the potential, heavy metal-based, ecological risk of *P. notoginseng* cultivation soil.

### 3.4. Characteristics of Heavy Metal Content in P. notoginseng Root of Different Cultivation Regions

The tuber of *P. notoginseng* is the main raw material in some Chinese patent medicine preparations. Plants readily assimilate elements from soil through roots. The content of heavy metals in plants’ roots directly affects the safety and quality of plant-based medicines [1]. Reports of above-average heavy metals in the *P. notoginseng* tuber have occasionally been made [4]. Therefore, a comprehensive understanding of heavy metal pollution in cultivation regions will help us avoid the contamination of *P. notoginseng*.

Table 9 shows the content of six different heavy metals in *P. notoginseng* rhizome samples, while Table 10 shows the number for each heavy metal that exceeds the safety standard. Pb, Cd, Cr, As, Hg, and Cu contents in *P. notoginseng* tuber samples ranged between 0.04–3.3, 0.04–0.33, 0.2–5.4, 0.1–1.8, 0.00–0.02, and 5.0–20.9 mg·kg^−1^, respectively. Average and median values of the 22 plant samples for six kinds of heavy metal were all lower than the safety standards established by the WM/T2-2004 [18] and Chinese Pharmacopoeia [20] methods. However, a few samples showed Cd, Cr, and Cu contents that exceeded the standards mentioned above [18,22]. The above-average rates were 13.6%, 22.7%, and 9.1%, respectively (in this order: Cr > Cd > Cu). The contents of Pb, As, and Hg in all plant samples were below the safety standard.

Safety regulations of various regions strictly manage the heavy metal content of traditional Chinese medicine preparations [22,36,37]. However, rare events of above-average heavy metal poisoning have occurred in the past [38,39,40,41]. Above-average contents of heavy metals in *P. notoginseng*, which is a primary raw material of many Chinese patent medicines, are also occasionally reported. The results of Lin et al. indicate that the concentrations of As and Pb in *P. notogiseng* exceed the limit standards by 56% and 97%, respectively [40]. The excessive degree of heavy metals’ content in *P. notoginseng* plants can be numbered as follows: As > Pb > Cr > Cd. Meanwhile, Hg content is within the safety standards’ limit [5,6,29]. Surprisingly, in our investigation the content of Hg, As, and Pb were all under legal limits [18,22]. In addition, contents of the six heavy metals we studied in *P. notoginseng* were lower than as shown in prior research. This might be caused by sampling time and sites, as well as drying method. The prior researches dried the samples with a drying oven [5,6,29] whereas we used air drying. However, the results obtained in the present study were consistent with the results of our previous study, where we stated that proper processing decreases the content of heavy metals. Moreover, we indicated that the content of Cd in the tuber only exceeds the standards when the roots do not go through appropriate processing [27].

### 3.5. Estimate Daily Intakes (EDI) of Heavy Metal, Target Hazard Quotients (THQs) and Hazard Index (HI) for Intake of Potential Health Risk Individual

Table 11 shows the estimated daily intakes (EDI) for *P. notoginseng* consumers exposed to heavy metals. The EDIs for adults were averaged similarly for women and men. The EDIs range of Pb, Cd, Cr, Hg, As, Hg, and Cu were 0.077–0.231, 0.006–0.019, 0.174–0.523, 0.040–0.120, 0.001–0.002, and 0.591–1.773 μg·kg^−1^·bw·day, all of which were significantly below the respective PTWI values recommended by international regulation bodies. The root of *P. notoginseng* is usually used in drug and health care products. EDIs of heavy metal through *P. notoginseng* consumption are not an important pathway for dietary exposure (rice, vegetables, fruits, fish, meat, eggs, and water) in the population. Thus, the EDIs of heavy metals through *P. notoginseng* consumption are much lower in practice.

THQs are also recognized as useful parameters for the evaluation of risk associated with the consumption of food contaminated with heavy metals. Table 11 lists the THQs of individual heavy metals, through *P. notoginseng* consumption, for humans. The range of the THQs of Pb, Cd, Cr, Hg, As, Hg, and Cu were 0.022–0.065, 0.008–0.023, 0.182–0.547, 0.013–0.040, 0.004–0.011, and 0.001–0.004 μg·kg^−1^·bw·day. No individual heavy metal THQ values exceeded the maximum recommendation value, suggesting that the population would not be confronted with a significant potential health risk by intake of Pb, Cd, Cr, Hg, As, Hg, and/or Cu through *P. notoginseng* consumption. The THQs of heavy metals from *P. notoginseng* consumption were in the order of Cr > Pb > Cd > Cu > Hg > As. It can be seen that Cr ingestion had the highest potential health risk of adverse effects, while ingestion of As had the minimum risk.

In the present study, the *HI* value was ranged from 0.230 to 0.689, and lower than the standard value of one. We demonstrated that ingestion of *P. notoginseng* produced in Yunnan would not result in overexposure of heavy metals. Thus, no adverse effect is posed to consumers’ health. We may conclude that there is only a low health risk, even with long-term intakes of *P. notoginseng* following the recommendation of the Chinese Pharmacopoeia [22]. The present results indicate that the relative contributions of Cr to the *HI* were over 85%, and were therefore the major contributions to the potential health risk of non-carcinogenic effects.

## 4. Conclusions

The soil samples analyzed from a variety of producing areas of *P. notoginseng* demonstrate that most of the *P. notoginseng* cultivation soil is polluted by As, Cd, and Hg, with a considerable level of risk. This proves that most of the *P. notoginseng* cultivation soil in the Yunnan province is characterized by slight and moderate ecological risk. Therefore, we must carefully consider the presence of As, Cd, and Hg when selecting cultivation land for *P. notoginseng*. The contents of Hg, As, and Pb we obtained were all under legal limits. EDIs of heavy metals through *P. notoginseng* consumption are not a significant pathway for dietary exposure of the population. No THQ values for an individual element exceed the value of one. The *HI* value we obtained indicated that there is a very low health risk even with long-term intake of *P. notoginseng* following the recommendations of the Chinese Pharmacopoeia.

## Figures and Tables

**Table 1:** Soil and plant sampling points of *P. notoginseng* cultivation in Yunnan, China.

Number	Sampling Point	Longitude	Latitude	Altitude
1	Miechang-Maguan	104.21	22.88	1648
2	Wanzizhai-Maguan	104.51	22.89	1332
3	Jiahanjing-Maguan	104.42	22.91	1533
4	Mabai-Maguan *	104.45	22.95	1325
5	Bazhaixiang-Maguan	104.08	22.98	1690
6	Xinxiaozhai-Maguan	104.42	22.98	1580
7	Luxi-Honghe	103.20	23.02	1677
8	Muyang-Funing *	106.32	23.13	762
9	Yangliujing-Wenshan	104.28	23.20	1467
10	Pingba-Wenshan *	104.13	23.25	1756
11	Benggu-Xichou *	104.63	23.38	1403
12	Mesa-Xichou	104.62	23.42	1510
13	Dongshan-Wenshan 1 *	104.23	23.43	1597
14	Dongshan-Wenshan 2	104.32	23.47	1580
15	Dongshan-Wenshan 3	104.23	23.49	1597
16	Guanting-Jianshui *	102.70	23.52	1975
17	Puxiong-Jianshui *	102.98	23.55	1898
18	Niujie-Shiping *	102.57	23.62	2094
19	Ganhe-Yanshan *	104.43	23.70	1479
20	Pingyuanjie-Yanshan *	103.73	23.75	1464
21	Guangnan-Wenshan *	104.88	23.77	1728
22	Jingping-Qiubei *	104.17	23.98	1481
23	Shuangying-Qiubei *	104.13	24.15	1449
24	Hongta-Yuxi *	102.43	24.42	1921
25	Luliang-Qujing	103.73	24.42	2021
26	Mile-Honghe *	103.25	24.43	2091
27	Yiliang-Kunming *	103.07	24.57	1973
28	Mengzi-Honghe	103.90	24.58	1935
29	Shilin-Kunming *	103.35	24.80	2130
30	Luoping-Qujing *	104.18	24.82	2034
31	Shizong-Qujing *	104.00	24.88	2034
32	Qilin-Qujing 1	104.05	25.17	1901
33	Qilin-Qujing 2	104.05	25.17	1896
34	Xiaoshao-Kunming *	102.97	25.18	1986
35	Baiyi-Kunming	102.85	25.23	1970
36	Fuyuan-Qujing *	104.26	25.43	1835
37	Xundian-Kunming *	103.33	25.73	2114

* Plant sampling points of *P. notoginseng*.

**Table 2:** Classification standards of soil heavy metal pollution evaluation.

Degree	$P_{i}$	Degree of Pollution	Pollution Level
1	$P_{i}$ ≤ 0.7	Safe	Clean
2	0.7 < $P_{i}$ ≤ 1	Warning level	Good
3	1 < $P_{i}$ ≤ 2	Slightly polluted	Soil and plant were polluted
4	2 < $P_{i}$ ≤ 3	Medium polluted	Soil and plant were moderate polluted
5	$P_{i}$ > 3	Heavy polluted	Soil and plant were heavy polluted

**Table 3:** Reference $C_{n}^{i}$ and toxic coefficient $T_{r}^{i}$ of different heavy metals.

Index	Pb	Cd	Cr	As	Hg	Cu
$C_{n}^{i}$ mg·kg^−1^	42.4	0.24	76.3	16.0	0.048	47.2
$T_{r}^{i}$	5	30	2	10	40	5

$C_{n}^{i}$, soil environment background value of Yunnan Province [21].

**Table 4:** Heavy metal content in soil samples (Mean ± SD, mg·kg^−1^).

Number	Pb	Cd	Cr	As	Hg	Cu
1	57.5 ± 2.9	0.21 ± 0.01	48.6 ± 2.4	60.9 ± 3.0	0.12 ± 0.01	48.2 ± 2.4
2	68.9 ± 3.1	0.22 ± 0.01	4.8 ± 0.2	96.8 ± 4.4	0.39 ± 0.02	65.3 ± 2.9
3	28.8 ± 1.3	0.23 ± 0.01	4.8 ± 0.2	10.0 ± 0.5	0.15 ± 0.01	43.2 ± 1.9
4	28.3 ± 1.3	0.29 ± 0.01	78.5 ± 3.5	47.2 ± 2.1	0.26 ± 0.01	47.9 ± 2.1
5	138.5 ± 6.0	0.24 ± 0.01	19.0 ± 0.8	5.5 ± 0.2	0.18 ± 0.01	25.4 ± 1.1
6	126.5 ± 5.3	0.29 ± 0.01	51.1 ± 2.1	29.9 ± 1.3	0.37 ± 0.02	43.1 ± 1.8
7	55.8 ± 3.4	0.18 ± 0.01	126.5 ± 7.6	77.2 ± 4.6	0.08 ± 0.01	18.3 ± 1.1
8	36.2 ± 2.1	0.21 ± 0.01	176.8 ± 10.4	50.4 ± 3.0	0.76 ± 0.05	24.6 ± 1.5
9	27.7 ± 1.2	0.12 ± 0.01	135.3 ± 5.7	4.1 ± 0.2	0.13 ± 0.01	15.2 ± 0.6
10	38.0 ± 1.1	0.22 ± 0.01	85.3 ± 2.6	20.5 ± 0.6	0.12 ± 0.00	37.9 ± 1.1
11	66.6 ± 1.4	0.75 ± 0.02	123.3 ± 2.6	57.5 ± 1.2	0.52 ± 0.01	30.3 ± 0.6
12	63.5 ± 2.2	0.71 ± 0.03	80.0 ± 2.8	73.7 ± 2.6	0.90 ± 0.03	44.4 ± 1.6
13	27.9 ± 1.3	0.13 ± 0.01	109.5 ± 5.0	43.4 ± 2.0	0.64 ± 0.03	27.3 ± 1.3
14	40.4 ± 1.9	0.40 ± 0.02	82.0 ± 3.9	59.6 ± 2.8	0.23 ± 0.01	13.4 ± 0.6
15	40.4 ± 2.0	0.43 ± 0.02	121.6 ± 6.1	65.6 ± 3.3	0.12 ± 0.01	46.56 ± 2.3
16	90.6 ± 5.4	0.55 ± 0.03	113.9 ± 6.8	94.1 ± 5.6	0.10 ± 0.01	44.7 ± 2.7
17	91.4 ± 4.8	0.22 ± 0.01	106.1 ± 5.6	126.3 ± 6.7	0.15 ± 0.01	22.8 ± 1.2
18	91.5 ± 4.9	0.77 ± 0.04	178.9 ± 9.5	119.2 ± 6.3	0.13 ± 0.01	25.7 ± 1.4
19	34.2 ± 1.5	0.28 ± 0.01	72.0 ± 3.2	22.5 ± 1.0	0.05 ± 0.00	31.3 ± 1.4
20	51.9 ± 3.5	0.25 ± 0.02	89.6 ± 6.1	108.0 ± 7.3	0.18 ± 0.01	20.6 ± 1.4
21	29.9 ± 2.1	0.06 ± 0.00	70.9 ± 5.0	40.1 ± 2.9	0.09 ± 0.01	62.7 ± 4.5
22	21.7 ± 0.4	0.62 ± 0.01	83.4 ± 1.5	208.5 ± 3.8	0.62 ± 0.01	36.2 ± 0.7
23	54.6 ± 1.2	0.29 ± 0.01	101.6 ± 2.1	75.4 ± 1.6	0.57 ± 0.01	42.8 ± 0.9
24	151.1 ± 8.2	0.26 ± 0.01	117.3 ± 6.3	27.0 ± 1.5	0.09 ± 0.01	49.6 ± 2.7
25	41.2 ± 0.9	0.29 ± 0.01	45.9 ± 1.0	56.1 ± 1.2	0.48 ± 0.01	13.2 ± 0.3
26	101.1 ± 5.7	0.29 ± 0.02	109.7 ± 6.1	25.5 ± 1.4	0.07 ± 0.01	29.1 ± 1.6
27	55.8 ± 1.4	0.49 ± 0.01	128.8 ± 3.2	21.4 ± 0.5	0.06 ± 0.00	37.8 ± 0.9
28	62.7 ± 1.4	0.48 ± 0.01	150.7 ± 3.5	85.5 ± 2.0	0.61 ± 0.014	42.6 ± 1.0
29	97.9 ± 3.2	0.290 ± 0.01	160.3 ± 5.3	13.4 ± 0.4	0.94 ± 0.03	48.8 ± 1.6
30	116.2 ± 5.0	0.47 ± 0.02	85.6 ± 3.7	84.9 ± 3.7	0.10 ± 0.00	55.6 ± 2.4
31	70.7 ± 0.9	0.23 ± 0.00	158.7 ± 2.1	74.0 ± 1.0	0.22 ± 0.00	37.7 ± 0.5
32	49.5 ± 2.6	0.62 ± 0.03	176.2 ± 9.3	86.0 ± 4.6	0.17 ± 0.01	41.8 ± 2.2
33	50.7 ± 2.2	0.91 ± 0.04	182.6 ± 7.9	80.4 ± 3.5	0.12 ± 0.01	40.0 ± 1.7
34	42.9 ± 1.0	0.28 ± 0.01	98.1 ± 2.3	20.7 ± 0.5	0.045 ± 0.001	25.2 ± 0.6
35	47.1 ± 1.6	0.23 ± 0.01	78.8 ± 2.6	26.7 ± 0.9	0.071 ± 0.002	17.7 ± 0.6
36	37.5 ± 1.6	0.21 ± 0.01	124.9 ± 5.4	5.3 ± 0.2	0.104 ± 0.004	10.1 ± 0.4
37	45.1 ± 1.4	0.37 ± 0.01	107.5 ± 3.4	8.4 ± 0.3	0.083 ± 0.003	31.5 ± 1.0

**Table 5:** Analysis of heavy metal contents in *P. notoginseng* cultivation soil (*n* = 37, mg·kg^−1^).

Index	Safety Standard	Min	Max	Mean	Median	SD	Exceeded Standard	
							No.	%
Pb	≤250	21.7	151.1	61.6	51.9	32.7	0	0.00
Cd	≤0.3	0.06	0.91	0.35	0.29	0.20	13	35.1
Cr	≤150	4.8	182.6	102.4	106.1	45.7	7	18.9
As	≤40	4.1	208.5	57.1	56.1	42.3	23	62.1
Hg	≤0.3	0.04	0.94	0.27	0.15	0.25	11	29.7
Cu	≤50	10.1	65.3	35.1	37.7	13.8	3	8.1

**Table 6:** Pollution status of *P. notoginseng* cultivation soil (*n* = 37).

Index	API	PIR	Classification of *P_i_*				
			$P_{i}$ ≤ 0.7	0.7 < $P_{i}$ ≤ 1	1 < $P_{i}$ ≤ 2	2 < $P_{i}$ ≤ 3	$P_{i}$ > 3
Pb	0.25	0.09–0.60	37	0	0	0	0
Cd	1.18	0.20–3.22	6	18	7	5	1
Cr	0.68	0.03–1.22	18	12	7	0	0
As	1.43	0.10–5.21	13	1	13	8	2
Hg	0.90	0.15–3.14	23	3	5	5	1
Cu	0.70	0.20–1.31	17	17	3	0	0
$P_{c}$	1.51	0.66–4.08	4	7	17	8	1

API, average pollution index; PIR, Pollution index range.

**Table 7:** Single heavy metal potential environment risk index ($E_{r}^{i}$) classification of *P. notoginseng* cultivation soil (*n* = 37).

Index	Min	Max	Mean	Sample Frequency Distribution				
				$E_{r}^{i}$ ≤ 40	40 < $E_{r}^{i}$ ≤ 80	80 < $E_{r}^{i}$ ≤ 160	160 < $E_{r}^{i}$ ≤ 320	$E_{r}^{i}$ > 320
				Slight	Middle	Strong	Very Strong	Extremely Strong
Pb	2.6	17.8	7.3	37	0	0	0	0
Cd	7.4	113.3	44.3	24	9	4	0	0
Cr	0.12	4.8	2.7	37	0	0	0	0
As	2.6	130.0	35.6	22	14	1	0	0
Hg	37.2	785.3	225.2	2	8	13	4	10
Cu	1.1	6.9	3.7	37	0	0	0	0

**Table 8:** *RI* classification of *P. notoginseng* cultivation soil.

Potential Ecological Risk Degree	Min	Max	Mean	Comprehensive Potential Environment Risk Index			
				*RI* ≤ 150 Slight	150 < *RI* ≤ 300 Moderate	300 < *RI* ≤ 600 Strong	*RI* > 600 Extremely Strong
*RI*	95.8	895.1	318.7	10	16	6	5
Proportion				27.0%	43.2%	16.2%	13.5%

**Table 9:** Heavy metals contents in rhizome of *P. notoginseng* (mean ± SD, mg·kg^−1^).

Number	Pb	Cd	Cr	As	Hg	Cu
1	2.42 ± 0.12	0.15 ± 0.01	1.98 ± 0.10	1.45 ± 0.07	0.015 ± 0.0008	10.61 ± 0.53
8	0.95 ± 0.04	0.06 ± 0.00	3.32 ± 0.14	0.82 ± 0.04	0.018 ± 0.0008	20.87 ± 0.90
10	2.80 ± 0.01	0.05 ± 0.00	4.51 ± 0.01	0.59 ± 0.00	0.004 ± 0.0001	13.41 ± 0.04
11	1.59 ± 0.07	0.17 ± 0.01	0.62 ± 0.03	1.49 ± 0.07	0.016 ± 0.0007	16.14 ± 0.73
13	1.92 ± 0.10	0.05 ± 0.00	1.27 ± 0.06	0.42 ± 0.02	0.016 ± 0.0008	10.5 ± 0.54
16	0.60 ± 0.03	0.23 ± 0.01	0.40 ± 0.02	1.60 ± 0.07	0.012 ± 0.0005	7.96 ± 0.36
17	0.27 ± 0.01	0.05 ± 0.00	0.22 ± 0.01	0.41 ± 0.02	0.010 ± 0.0005	6.33 ± 0.33
18	0.20 ± 0.01	0.06 ± 0.00	1.94 ± 0.07	0.49 ± 0.02	0.008 ± 0.0003	8.14 ± 0.28
19	3.26 ± 0.14	0.08 ± 0.00	1.42 ± 0.06	0.70 ± 0.03	0.011 ± 0.0005	12.42 ± 0.53
20	1.25 ± 0.06	0.07 ± 0.00	1.72 ± 0.08	0.46 ± 0.02	0.008 ± 0.0004	19.52 ± 0.90
21	1.91 ± 0.13	0.05 ± 0.00	1.50 ± 0.10	0.57 ± 0.04	0.016 ± 0.0011	8.90 ± 0.60
22	2.76 ± 0.09	0.32 ± 0.01	1.76 ± 0.06	1.83 ± 0.06	0.012 ± 0.0004	10.31 ± 0.35
23	2.39 ± 0.09	0.05 ± 0.00	0.63 ± 0.02	1.02 ± 0.04	0.008 ± 0.0003	14.68 ± 0.53
24	0.31 ± 0.01	0.04 ± 0.00	0.85 ± 0.02	0.24 ± 0.01	0.016 ± 0.0004	6.61 ± 0.17
26	0.86 ± 0.02	0.33 ± 0.01	0.28 ± 0.01	0.66 ± 0.02	0.006 ± 0.0002	15.44 ± 0.45
27	0.04 ± 0.00	0.05 ± 0.00	1.63 ± 0.08	0.45 ± 0.02	0.007 ± 0.0003	10.53 ± 0.49
29	0.67 ± 0.03	0.04 ± 0.00	1.28 ± 0.07	0.31 ± 0.02	0.008 ± 0.0004	6.26 ± 0.32
30	2.24 ± 0.10	0.33 ± 0.02	4.11 ± 0.18	1.60 ± 0.07	0.009 ± 0.0004	12.78 ± 0.58
31	2.36 ± 0.08	0.06 ± 0.00	5.41 ± 0.18	1.73 ± 0.06	0.009 ± 0.0003	7.88 ± 0.27
34	1.24 ± 0.04	0.21 ± 0.01	1.75 ± 0.06	0.33 ± 0.01	0.008 ± 0.0003	4.95 ± 0.17
36	2.19 ± 0.04	0.04 ± 0.00	0.40 ± 0.01	0.10 ± 0.00	0.011 ± 0.0002	15.84 ± 0.29
37	1.64 ± 0.06	0.30 ± 0.01	3.76 ± 0.13	0.29 ± 0.01	0.007 ± 0.0002	20.04 ± 0.70

**Table 10:** Analysis of heavy metal contents in *P. notoginseng* rhizome (*n* = 22, mg·kg^−1^).

Heavy Metals	Safety Standard *	Min	Max	Mean	SD	Median	Exceeded Standard	
							No.	%
Pb	≤5	0.04	3.26	1.54	0.95	1.62	0	0
Cd	≤0.3	0.04	0.33	0.13	0.11	0.06	3	13.6
Cr	≤2	0.22	5.41	1.85	1.45	1.56	5	22.7
As	≤2	0.10	1.83	0.80	0.55	0.58	0	0
Hg	≤0.2	0.00	0.02	0.01	0.00	0.01	0	0
Cu	≤20	4.95	20.87	11.82	4.64	10.57	2	9.1

* Safety standards are referred to WM/T2-2004 [18] for Pb, Cd, As, Hg and Cu, while Cr is referred to China Pharmacopoeia [22].

**Table 11:** Estimate daily intakes (EDI, μg·kg^−1^·bw·day), target hazard quotients (THQs) and hazard index (*HI*) for intake of potential health risk individual from *P. notoginseng*.

	Pb	Cd	Cr	As	Hg	Cu	*HI*
EDI	0.077–0.231	0.006–0.019	0.174–0.523	0.040–0.120	0.001–0.002	0.591–1.773	-
THQ	0.022–0.065	0.008–0.023	0.182–0.547	0.013–0.040	0.004–0.011	0.001–0.004	0.230–0.689

## References

[1] Yan X.L., Liao X.Y., Yu B.B., Zhang W.B. (2011). Accumulation of soil arsenic by *Panax notoginseng* and its associated health risk. Chin. J. Environ. Sci..

[2] Nnorom I.C. (2014). Heavy metal contamination of herbal medicinal products and cosmetics: A course for concern. Der Pharm. Sin..

[3] Ernst E., Thompson C.J. (2001). Heavy metals in traditional Chinese medicines: A systematic review. Clin. Pharmacol. Ther..

[4] Zhu M.L., Yang J., Cui B., Jiang Y.X., Cao H.B. Health risks of cadmium in traditional Chinese herbal medicine *Panax Notoginseng* in Yunnan authentic region. Proceedings of the 5th Annual Meeting of Risk-Analysis-Council-of-China-Association-for-Disaster-Prevention.

[5] Yan X.L., Lin L.Y., Liao X.Y., Zhang W.B. (2012). Arsenic accumulation and resistance mechanism in *Panax notoginseng*, a traditional rare medicinal herb. Chemosphere.

[6] Yan X.L., Lin L.Y., Liao X.Y., Zhang W.B., Wen Y. (2013). Arsenic stabilization by zero-valent iron, bauxite residue, and zeolite at a contaminated site planting *Panax notoginseng*. Chemosphere.

[7] Song W., Chen B.M., Liu L. (2013). Soil heavy metal pollution of cultivated land in China. Res. Soil Water Conserv..

[8] Wang H.H., Yan T.T. (2006). Present situation of farmland soil and crop accumulation conditions in Yunnan province. Semin. Nat. Farml. Soil Pollut. Monit. Eval. Technol..

[9] Li Z., Ma Z., Kuijp T.J., Yuan Z., Huang L. (2014). A review of soil heavy metal pollution from mines in China: Pollution and health risk assessment. Sci. Total Environ..

[10] Samera H.H., James J.S., Martin M.S., Esam A.A., Pamela S.S., Jongbae H. (2014). Risk assessment of total and bioavailable potentially toxic elements (PTEs) in urban soils of Baghdad-Iraq. Sci. Total Environ..

[11] Neilson S., Rajakaruna N. (2015). Phytoremediation of agricultural soils: using plants to clean metal-contaminated arable land. Phytoremediation.

[12] Gall J.E., Boyd R.S., Rajakaruna N. (2015). Transfer of heavy metals through terrestrial food webs: A review. Environ. Monit. Assess..

[13] Sheng J.J., Wang X.P., Gong P., Tian L.D., Yao T.D. (2012). Heavy metals of the Tibetan top soils. Environ. Sci. Pollut. Res..

[14] Pytlakowska K., Kita A., Janoska P., Połowniak M., Kozik V. (2012). Multi-element analysis of mineral and trace elements in medicinal herbs and their infusions. Food Chem..

[15] Zheng N., Wang Q.C., Zhang X.W., Zheng D.M., Zhang Z.S., Zhang S. (2007). Population health risk due to dietary intake of heavy metals in the industrial area of Huludao City, China. Sci. Total Environ..

[16] United States Environmental Protection Agency (1986). Guidelines for the health risk assessment of chemical mixtures. Fed. Regist..

[17] National Environmental Protect Bureau of China and National Technology Supervise Bureau of China (1995). Chinese Standards: Chinese Standards. Environmental Quality Standard for Soils. GB15618-1995.

[18] Ministry of Commerce of the People’s Republic of China (2004). Green Standards of Medicinal Plants and Preparations for Foreign Trade and Economy, WM/T2-2004.

[19] Hankson L. (1980). An ecology risk index for aquatic pollution control: A sedimentological approach. Water Res..

[20] Yan X.D., Gao D., Zhang F., Zeng C., Xiang W., Zhang M. (2013). Relationships between heavy metal concentrations in roadside topsoil and distance to road edge based on field observations in the Qinghai-Tibet Plateau, China. Int. J. Envion. Res. Public Health.

[21] Shi J., Zhang N.M. (2010). The distributing character of heavy metals and its pollution estimate in greenhouse soils of Yunnan Province. J. Yunnan Agric. Univ..

[22] National Pharmacopoeia Committee of the People’s Republic of China (2015). China Pharmacopoeia.

[23] United States Environmental Protection Agency (2000). Risk-Based Concentration Table.

[24] Chien L.C., Huang T.C., Choang K.Y., Yeh C.Y., Meng P.J., Shieh M.J., Ha B.C. (2002). Daily intake of TBT, Cu, Zn, Cd and As for fishermen in Taiwan. Sci. Total Environ..

[25] Food and Agriculture Organization/World Health Organization, FAO/WHO, Geneva. http://apps.who.int/food-additives-contaminants-jecfa-database/search.aspx.

[26] Harrison R.M., Chirgawi M.B. (1989). The assessment of air and soil as contributors of some trace metals to vegetable plants III-experiments with field-grown plants. Sci. Total Environ..

[27] Liu D.H., Xu N., Wang L., Cui X.M., Guo L.P., Zhang Z.H., Wang J.J., Yang Y. (2014). Effects of different cleaning treatments on heavy metal removal of *Panax notoginseng* (Burk) F. H. Chen. Food Addit. Contam. A.

[28] Zu Y.Q., Su J.J., Guo X.H., Min Q., Feng G.Q., Wu J., Yang L.Y., Li Y. (2014). Effects of As spatial distribution and physical and chemical characteristics of soil on As contents in *Panax notoginseng* in Wenshan plantation area. Ecol. Environ. Sci..

[29] Chen L., Mi Y., Lin X., Liu D., Zeng M., Chen X. (2014). Investigation and analysis of heavy metal pollution related to soil-*Panax notoginseng* system. Chin. J. Chin. Mater. Med..

[30] Lin L.Y., Yan X.L., Liao X.Y., Zhang Y.X. (2014). Accumulation of soil Cd, Cr, Cu, Pb by *Panax notoginseng* and its associated health risk. Acta Ecol. Sin..

[31] Liu J.B., Lu Y.N., Zou T. (2012). The analysis and evaluation on heavy metal pollution of topsoil in Chinese large-scale cities. Energy Procedia.

[32] Li W.X., Zhang X.X., Wu B., Sun S.L., Chen Y.S., Pan W.Y., Zhao D.Y., Cheng S.P. (2008). A comparative analysis of environmental quality assessment methods for heavy metal-contaminated soils. Pedosphere.

[33] Wu Y.G., Xu Y.N., Zhang J.H., Hu S.H. (2010). Evaluation of ecological risk and primary empirical research on heavy metals in polluted soil over Xiaoqinling gold mining region, Shanxi, China. Trans. Nonferr. Met. Soc..

[34] Guo W.H., Liu X.B., Liu Z.G., Li G.F. (2010). Pollution and potential ecological risk evaluation of heavy metals in the sediments around Dongjiang harbor, Tianjin. Procedia Environ. Sci..

[35] Min X.B., Xie X.D., Chai L.Y., Liang Y.J., Li L., Ke Y. (2013). Environmental availability and ecological risk assessment of heavy metals in zinc leaching residue. Trans. Nonferr. Met. Soc..

[36] The United States Pharmacopeia Convention (2012). United States Pharmacopeia 35.

[37] Ministry of Health, Labor and Welfare, Prefectural office in Japan (2011). The Pharmacopoeia of Japan.

[38] Obi E., Dora N., Akunyili B.E., Orish E.O. (2006). Heavy metal hazards of Nigerian herbal remedies. Sci. Total Environ..

[39] Street R.A. (2012). Heavy metals in medicinal plant products—An African perspective. S. Afr. J. Bot..

[40] Lin L.Y., Yu B.B., Liao X.Y., Yan X.L., Zhang Y.X. (2013). Contents and health risk of As and heavy metals in *Panax notoginseng* and their pharmaceutical preparations. Asia J. Ecotoxicol..

[41] Ernst E. (2002). Toxic heavy metals and undeclared drugs in Asian herbal medicines. Trends Pharmacol. Sci..

